# Physiological Thresholds and Adaptation Mechanisms of the Ili Perch (*Perca schrenkii*) to Chloride-Type Saline Water

**DOI:** 10.3390/ani16010063

**Published:** 2025-12-25

**Authors:** Kaipeng Zhang, Shixin Gao, Guanping Xing, Yichao Hao, Zhulan Nie, Jie Wei, Tao Ai, Shijing Zhang, Jiasong Zhang, Zhaohua Huang

**Affiliations:** 1 College of Life Science and Technology, Tarim University, Alar 843300, China; zkp19980604@163.com (K.Z.); follow_spot@163.com (S.G.); xingguanping1111@163.com (G.X.); 10757242166@stumail.taru.edu.cn (Y.H.); 2 Xinjiang Production & Construction Corps Key Laboratory of Protection and Utilization of Biological Resources in Tarim Basin, Alar 843300, China; 3 Xinjiang Production and Construction Corps Aquaculture Technology Promotion General Station, Urumqi 830002, China; taoai202506@163.com; 4 Yutian Fengze Technology Aquatic Products Co., Ltd., Hetian 848400, China; 17797917561@163.com (S.Z.); jiasongzhang@hotmail.com (J.Z.); 5 Aral Changxin Fishery Co., Ltd., Aral 843300, China; huangzhaohua1968@163.com

**Keywords:** salinity tolerance, osmoregulation, oxidative stress, immune response, sustainable aquaculture

## Abstract

Global freshwater scarcity necessitates the exploitation of alternative water resources for aquaculture. Chloride-type saline-alkaline water, characterized by high salinity but moderate pH, is widely distributed in arid regions worldwide and represents a vast, underutilized resource. This study comprehensively evaluated the potential of the endemic Ili perch (*Perca schrenkii*) for aquaculture in such environments. Through acute stress experiments, we determined its **96** h median lethal salinity (LC_50_) to be **12.396** ppt, with a safe concentration of **3.72** ppt. Physiological analysis revealed a critical salinity threshold of **13** ppt, beyond which osmoregulatory collapse (indicated by plasma Na^+^/K^+^ dysregulation and Na^+^-K^+^-ATPase suppression), oxidative damage (elevated malondialdehyde), and immune suppression occurred. In contrast, chronic **60**-day exposure to salinities up to 7 ppt demonstrated successful long-term acclimation. Acclimated fish re-established ion homeostasis, as plasma ion levels normalized, and exhibited sustained antioxidant enzyme (SOD, CAT) and immune parameter (AKP, ACP, IgM) activities without signs of damage. Our findings establish *P. schrenkii* as a highly promising species for chloride-type saline-alkaline aquaculture. The study provides a physiological framework for its adaptation and offers evidence-based salinity guidelines for its sustainable cultivation, thereby contributing to the expansion of aquaculture into non-traditional water resources.

## Simple Summary

Global freshwater scarcity has pushed the exploration of saline-alkaline aquaculture, especially in Northwest China where chloride-type saline-alkaline water is abundant but underused. *Perca schrenkii* (Ili perch), an endemic fish in the Ili River basin, shows promise for such aquaculture, yet its salinity tolerance and adaptation ability remain unclear. This study involved two experiments: an acute high-salinity test (**11–15** ppt, **96** h) and a chronic low-salinity acclimation test (**3–7** ppt, **60** days). We found the **96** h median lethal salinity (LC_50_) of *P. schrenkii* was **12.396** ppt, and it could fully adapt to long-term culture in ≤7 ppt salinity—with 3 ppt causing almost no stress. These results provide clear salinity guidelines for the safe and sustainable cultivation of *P. schrenkii* in saline waters.

## 1. Introduction

Global warming, coupled with escalating water demand driven by population growth and socioeconomic development, has significantly exacerbated pressures on the planet’s limited freshwater resources. This challenge is particularly pronounced in water-scarce or water-stressed regions, where unmet water demand increasingly outstrips available renewable supplies—ultimately leading to a state of “water bankruptcy.” Such a scenario is accompanied by concomitant adverse socioeconomic consequences, including disruptions to agricultural productivity, constraints on industrial development, compromised public health services, and heightened risks of intersectoral or transboundary conflicts over water allocation [1]. This crisis is exacerbated by the rapid expansion of aquaculture: global aquatic animal production from aquaculture reached **87.5** million tonnes in **2023**, surpassing that of capture fisheries for the first time and intensifying pressure on limited freshwater reserves [2]. The growing dependence of agricultural and aquacultural production on the unsustainable exploitation of finite freshwater resources has further intensified pressures on global water security. Consequently, the need to explore alternative water resources tailored for aquacultural practices has become increasingly imperative [3].

China, the world’s largest aquaculture producer, confronts unique challenges: freshwater aquaculture constitutes over **75**% of its total aquatic production [4], yet the country holds only 6% of global freshwater reserves, with extreme regional disparities. Northwest China, a core arid-agricultural zone, harbors approximately 30 million hectares of exploitable saline-alkaline water, of which chloride-type saline-alkaline water (dominated by Cl^−^·Na^+^ ions, total dissolved solids [TDS] **5–30** g/L, pH **7.2–9.1**) accounts for ~**60**% [5]. Developing these underutilized resources offers a transformative solution: saline-alkaline water aquaculture not only alleviates freshwater competition but also reduces soil salinity through nutrient cycling, achieving ecological and economic co-benefits [6,7]. This water type is geochemically distinct from carbonate-type saline-alkaline water (characterized by HCO_3_^−^/CO_3_^2−^-induced pH stress, pH > **9.0**), as its primary physiological challenge to freshwater fish is osmotic imbalance driven by high salinity [8,9,10,11,12,13]. Osmotic stress disrupts ion homeostasis (e.g., Na^+^/K^+^ dysregulation) [14], triggers reactive oxygen species (ROS) accumulation [15], and impairs innate immune response [16]—all of which reduce survival and growth in aquaculture settings [15,17].

A critical prerequisite for successful saline-alkaline water aquaculture is identifying fish species with inherent tolerance to elevated salinity, as freshwater fish typically suffer osmotic imbalance, oxidative stress, and growth inhibition under high-salt conditions [18,19]. Against this backdrop, *Perca schrenkii* (Ili perch)—an endemic carnivorous fish of the Ili River basin in Xinjiang—emerges as a uniquely promising candidate for chloride-type saline-alkaline aquaculture. Pilot field observations and genetic studies have documented its presence in oligohaline reaches of the Ili River delta (salinity up to 3 ppt), a niche where sympatric freshwater species (e.g., *Ctenopharyngodon idella*, *Cyprinus carpio*) are rarely detected—suggesting intrinsic evolutionary adaptations to mild salinity [20,21]. Moreover, recent studies on Xinjiang’s endemic fish have shown that mild saline exposure (**3–5** ppt) can enhance muscle nutritional quality, suggesting similar potential for *P. schrenkii* [22,23]. This combination of ecological adaptability and potential for quality enhancement positions it as an ideal model to unravel the mechanisms of fish adaptation to chloride-type saline-alkaline water.

Notably, while the saline tolerance of its congener *Perca fluviatilis* (Eurasian perch) has been well-characterized—with freshwater populations exhibiting a **96** h median lethal concentration (LC_50_) of **10** ppt and growth inhibition above 8 ppt [24,25,26]—critical knowledge gaps persist for *P. schrenkii*. First, its acute tolerance threshold to chloride-type saline stress (e.g., NaCl-simulated osmotic imbalance) remains unquantified, precluding the identification of safe salinity ranges for aquaculture. Second, the tissue-specific dynamics of key adaptive pathways—including gill/kidney osmoregulation, hepatic oxidative defense, and intestinal/plasma immunity—under both acute and chronic salinity exposure are unknown. For example, Na^+^-K^+^-ATPase is critical for osmotic balance, but its activation kinetics in *P. schrenkii* under chloride-type stress have never been measured. Third, no studies have evaluated its long-term growth performance in sub-lethal chloride-type salinities—a prerequisite for translating physiological data to commercial aquaculture practice [27].

To address these gaps, this study designed controlled experiments using NaCl to simulate the core stress of chloride-type saline-alkaline water (osmotic imbalance), thereby isolating its effects from other confounding factors. We conducted two complementary experiments: (1) an acute salinity stress experiment (salinity gradients: **11**, **12**, **13**, **14**, **15** ppt; sampling times: 0, 6, **12**, **24**, **48**, **96** h) to define the **96** h LC_50_ and characterize short-term behavioral and physiological responses and (2) a chronic salinity stress experiment (salinity gradients: 0 [control], 3, 5, 7 ppt; sampling times: **30**, **60** days) to evaluate growth performance and long-term physiological homeostasis. The overarching goal was to establish a mechanistic framework for *P. schrenkii*’s adaptation to chloride-type saline-alkaline water, providing evidence-based recommendations for its sustainable cultivation. Additionally, this study aims to advance the broader understanding of freshwater fish adaptation to osmotic stress, offering a template for future research on other candidate species for saline-alkaline aquaculture.

## 2. Materials and Methods

### 2.1. Experimental Fish and Acclimation

Juvenile *Perca schrenkii* were collected from the Emin River basin (Xinjiang, China), a natural habitat of this species. Prior to experiments, fish were screened for health status (no injuries, uniform size, active swimming), with a final selected size of body length **12.15 ± 2.32** cm and body weight **7.53 ± 1.68** g.

Acclimation was conducted in **1000** L recirculating aquaculture systems (Zhongke Ruiying Aquaculture Equipment Co., Ltd., Wuxi, Jiangsu, China) for **14** days to minimize stress from transportation and environmental change. Acclimation conditions were maintained as follows: water temperature (**22 ± 1**) °C, pH **7.60 ± 0.35**, dissolved oxygen (**7.80 ± 0.52**) mg/L, ammonia nitrogen ≤ **0.3** mg/L, nitrite ≤ **0.03** mg/L, and salinity **0.65 ± 0.30** ppt (consistent with the Fishery Water Quality Standard, GB 11607-1989 [28]). Fish were fed a commercial extruded feed (crude protein ≥ **40**%, crude lipid ≥ 8%; Tongwei Co., Ltd., Chengdu, Sichuan, China) twice daily (**09:00** and **17:00**) at a feeding rate of 3% body weight. Feeding was withheld **24** h before the start of experiments to avoid gastrointestinal contents interfering with tissue sampling.

All experimental procedures were approved by the Animal Research Ethics Committee of Tarim University (Approval No. PB20250618003) and complied with the guidelines for the use of experimental animals in China.

### 2.2. Experimental Design

Two complementary experiments (acute and chronic salinity stress) were designed using NaCl (analytical grade, ≥**99.5**%, Nanjing, Jiangsu Chemical Reagent Co., Ltd., Nanjing, China) to simulate the core osmotic stress of chloride-type saline-alkaline water, with salinity stabilized for **24** h before experiments and re-measured using a Leici ZD-2 salinometer (Shanghai Yidian Scientific Instruments Co., Ltd., Shanghai, China) to ensure accuracy.

#### 2.2.1. Acute Salinity Stress Experiment

Based on preliminary experiments (no mortality at salinity < **11** ppt, **100**% mortality within **24** h at salinity > **15** ppt), 5 salinity gradients (**11**, **12**, **13**, **14**, **15** ppt) and 1 freshwater control group (0 ppt) were set. Each group included 3 biological replicates, with **20** fish per replicate (housed in **50** L glass tanks, Taizhou Kelin Experimental Equipment Co., Ltd., Taizhou, Zhejiang, China; with micro-aeration, dissolved oxygen ≥ 6 mg/L).

Experimental duration was **96** h, with sampling time points at 0, 6, **12**, **24**, **48**, and **96** h. At each time point, two types of measurements were conducted:

(1) Behavioral observation, mortality recording and quantification

Behavioral responses were recorded to characterize the concentration- and time-dependent stress effects, with 3 randomly selected fish per replicate (9 fish per group) tracked for **10** min per observation (to avoid transient errors).

Death was defined as no response to glass rod stimulation (touching the caudal peduncle), cessation of gill movement, and permanent sinking to the tank bottom. Mortality was recorded every 2 h to calculate survival rates for each group.

Key behavioral indicators, their measurement methods, and quantification standards are detailed in Table 1.

(2) Tissue and plasma sampling

After behavioral recording, 3 fish per replicate (9 fish per group) were anesthetized with **100** mg/L MS-222 (Sigma-Aldrich, St. Louis, MO, USA). Blood was collected from the caudal vein using heparinized syringes (1 mL, Jiangsu Kangjian Medical Devices Co., Ltd., Nantong, Jiangsu, China), centrifuged at **3000** rpm for 10 min at 4 °C to separate plasma, and stored at −**80** °C. Gill, liver, intestine, and kidney tissues were rapidly dissected, rinsed with pre-cooled physiological saline (**0.85**% NaCl, Nanjing Chemical Reagent Co., Ltd., Nanjing, Jiangsu, China) to remove blood and debris, snap-frozen in liquid nitrogen, and stored at −**80** °C for subsequent biochemical analyses.

#### 2.2.2. Chronic Salinity Stress Experiment

Considering the acute tolerance threshold and the actual salinity range of chloride-type saline-alkaline water in Northwest China (**3–10** ppt), 4 salinity gradients (0 [control], 3, 5, 7 ppt) were set. Each group included 3 biological replicates, with **30** fish per replicate (housed in **200** L glass tanks, daily water exchange rate **30**% to maintain water quality).

Experimental duration was **60** d, with sampling time points at **60** d. At each time point, tissue and plasma sampling measurements were conducted:

Sampling methods were consistent with the acute experiment (3 fish per replicate), with tissues and plasma stored at −**80** °C for biochemical analyses.

During both experiments, the photoperiod was maintained at **12**L:**12**D (08:00–20:00 light), and water quality parameters (temperature, pH, dissolved oxygen) were monitored daily using a multi-parameter water quality analyzer (YSI ProPlus, YSI Inc., Yellow Springs, OH, USA). Feces were siphoned under dim light every **24** h to avoid water pollution.

### 2.3. Determination of Physiological and Biochemical Indicators

All indicators were measured using commercial kits (Nanjing Jiancheng Bioengineering Institute, Nanjing, Jiangsu, China) following the manufacturers’ protocols, with instrument models and detection principles specified below in Table 2. For tissue sample preparation: Tissues were homogenized in a liquid nitrogen-precooled mortar with 9 volumes of ice-cold physiological saline (*w*/*v* = **1:9**). Homogenates were centrifuged at **3000** rpm for **10** min at 4°C, and the supernatant was diluted 5-fold with physiological saline before analysis. Plasma samples were used directly after centrifugation without dilution.

### 2.4. Data Processing and Statistical Analysis

#### 2.4.1. Acute Toxicity Parameters

Mortality rates at each time point were used to calculate the **96** h median lethal concentration (LC_50_) and **95**% confidence interval (CI) via Probit regression in IBM SPSS Statistics **26.0**. The safe concentration (SC) was calculated using the modified Kou’s method for freshwater fish [29]:

SC = **48** h LC_50_ × **0.3**/(**24** h LC_50_/**48** h LC_50_)^2^ (1)

#### 2.4.2. Behavioral and Physiological Data

Behavioral data: Graded indicators were converted to numerical values (**1–4**) for statistical analysis; count/rate indicators were expressed as “mean ± standard error (mean ± SE)”. Physiological and growth data: All data were expressed as “mean ± SE”. Statistical analyses were conducted using IBM SPSS Statistics **26.0**, and *p* < **0.05** was considered statistically significant.

Prior to analysis, normality (Shapiro–Wilk test) and homoscedasticity (Levene test) were verified. Two-way analysis of variance (Two-way ANOVA) was used to evaluate the main effects of salinity (fixed factor) and treatment time (fixed factor), as well as their interaction. If significant differences were detected (*p* < **0.05**), Tukey’s post hoc test was performed for pairwise comparisons.

All charts (line graphs for temporal dynamics, bar graphs for salinity comparisons, heatmaps for comprehensive behavioral responses) were generated using Origin **2023** (OriginLab Corporation, Northampton, MA, USA). Significant differences were marked by lowercase letters (*p* < **0.05**) for comparisons among salinity groups at the same time point, and uppercase letters (*p* < **0.05**) for comparisons among time points within the same salinity group.

## 3. Results

### 3.1. Effects of Acute Salinity Stress on Perca schrenkii

#### 3.1.1. Behavioral Responses to Acute Salinity Stress

**Figure 1:**
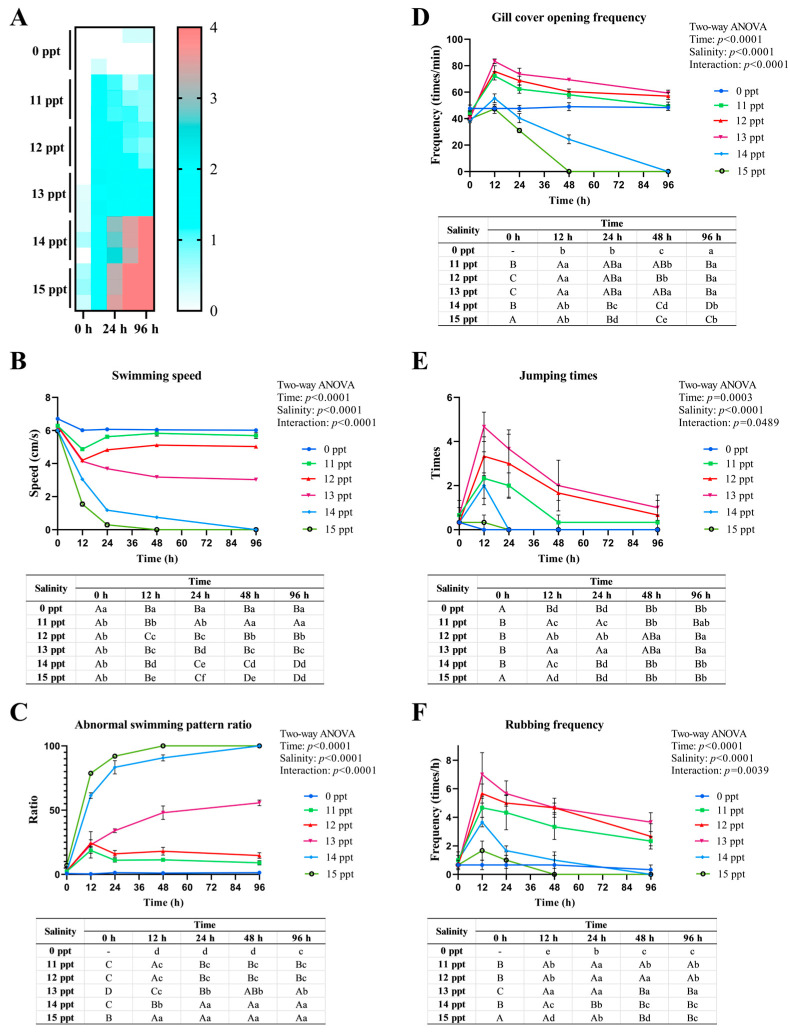
Behavioral responses of *Perca schrenkii* under acute salinity stress. (**A**) Heatmap of comprehensive behavioral abnormality score, with the color gradient (blue to red) representing the score range from **0** (no abnormality) **to 4** (severe abnormality). (**B**) Temporal dynamics of swimming speed (cm/s). (**C**) Temporal dynamics of abnormal swimming pattern ratio (%). (**D**) Temporal dynamics of gill cover opening frequency (times/min). (**E**) Temporal dynamics of jumping times (times/h). (**F**) Temporal dynamics of rubbing frequency (times/h). For (**B**–**F**), two-way ANOVA was used to analyze the effects of time, salinity, and their interaction, with significant differences marked by lowercase letters (*p* < **0.05**) for comparisons among salinity groups at the same time point, and uppercase letters (*p* < **0.05**) for comparisons among time points within the same salinity group.

Acute salinity stress induced distinct concentration- and time-dependent behavioral changes in *P. schrenkii*, with the severity of abnormalities escalating synergistically with increasing salinity and exposure duration (Figure 1). The heatmap integrating five behavioral indicators into a comprehensive abnormality score (ranging from 0 = no abnormality to 4 = severe abnormality) visually delineated three response zones: a safe adaptation zone (score < **1.0**) in the control group and partially in **11–12** ppt groups at later time points, a critical compensation zone (**1.0** ≤ score < **3.0**) primarily in **12–13** ppt groups, and a failure zone (score ≥ **3.0**) evident in **14–15** ppt groups from **12** h onward (Figure 1A). Regarding swimming speed, the 0 ppt group maintained stability (**6.0–9.0** cm/s), while salinity groups ≤ **12** ppt showed transient decreases at **6–12** h followed by recovery, the salinity group at **13** ppt exhibited a sustained decline, and groups ≥ **14** ppt rapidly deteriorated to near-static speed (Figure 1B). The abnormal swimming pattern ratio was low in the control group, increased transiently in ≤**12** ppt groups, rose continuously in **13** ppt, and surged to >**80**% in ≥**14** ppt groups by **12** h (Figure 1C). Gill cover opening frequency was stable in the control group, transiently elevated in ≤**12** ppt groups, sustained at high levels in **13** ppt, and showed a biphasic trend (peak then plummet) in ≥**14** ppt groups (Figure 1D). Jumping times were rare in the control group, peaked transiently in ≤**12** ppt groups, remained high in **13** ppt, and decreased rapidly to 0 in ≥**14** ppt groups after **24** h (Figure 1E). Rubbing frequency followed a similar pattern, with infrequent rubbing in the 0 ppt group, transient increases in ≤**12** ppt groups, sustained high frequency in **13** ppt, and sharp decreases to near-zero in ≥**14** ppt groups by **24** h (Figure 1F). Collectively, these behaviors exhibited a three-phase response: initial compensation (**6–12** h, low-salinity groups), sustained adaptation or critical compensation (**24–48** h, **13** ppt), and irreversible failure (≥**24** h, ≥**14** ppt), with **13** ppt emerging as a critical threshold separating reversible from irreversible behavioral deterioration.

#### 3.1.2. Mortality and Lethal Threshold (LC_50_)

**Figure 2:**
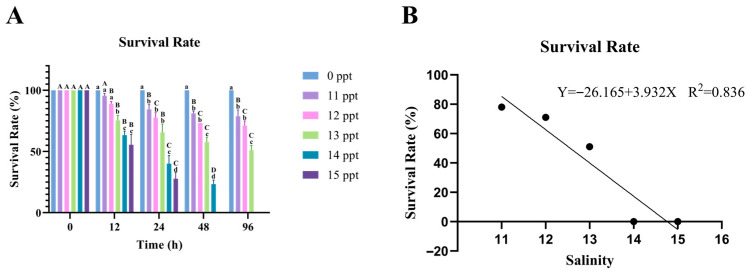
Mortality and lethal threshold of *Perca schrenkii* under acute salinity stress. (**A**) Temporal dynamics of survival rate (%) across different salinity groups (0, **11**, **12**, **13**, **14**, and **15** ppt). (**B**) Probit regression plot for **96** h mortality, where the *X*-axis represents salinity (ppt), the *Y*-axis represents survival rate (%). In (**A**), significant differences are marked by lowercase letters *p* < **0.05** for comparisons among salinity groups at the same time point, and uppercase letters *p* < **0.05** for comparisons among time points within the same salinity group.

Acute salinity stress induced significant time- and concentration-dependent mortality in *P. schrenkii*, with survival rates plummeting drastically in groups exposed to salinity ≥ **14** ppt (Figure 2A). To quantify the lethal threshold, a Probit regression analysis was performed on the **96** h survival data, yielding a **96** h median lethal concentration (LC_50_) of **12.396** ppt (Figure 2B). The regression equation (Y = −**26.165** + **3.932**X) exhibited a coefficient of determination R^2^ = **0.836**, indicating a reasonable fit of the model to the mortality data.

The LC_50_ value denotes that **50**% of *P. schrenkii* individuals would succumb to salinity stress at **12.396** ppt within **96** h. Correspondingly, the safe concentration (SC) was determined to be **3.7188** ppt. This SC represents a salinity level where *P. schrenkii* can maintain long-term survival without significant mortality risk.

Notably, the **13** ppt group exhibited an **82–85**% survival rate at **96** h (Figure 2A), which aligns with the LC_50_-derived threshold—salinity ≤ **12.396** ppt falls within the safe adaptation zone with negligible mortality risk, while salinity ≥ **14** ppt enters the lethal zone with >**50**% mortality by **96** h.

#### 3.1.3. Physiological and Biochemical Responses to Acute Salinity Stress

##### Osmotic Regulation of Acute Salinity Stress

**Figure 3:**
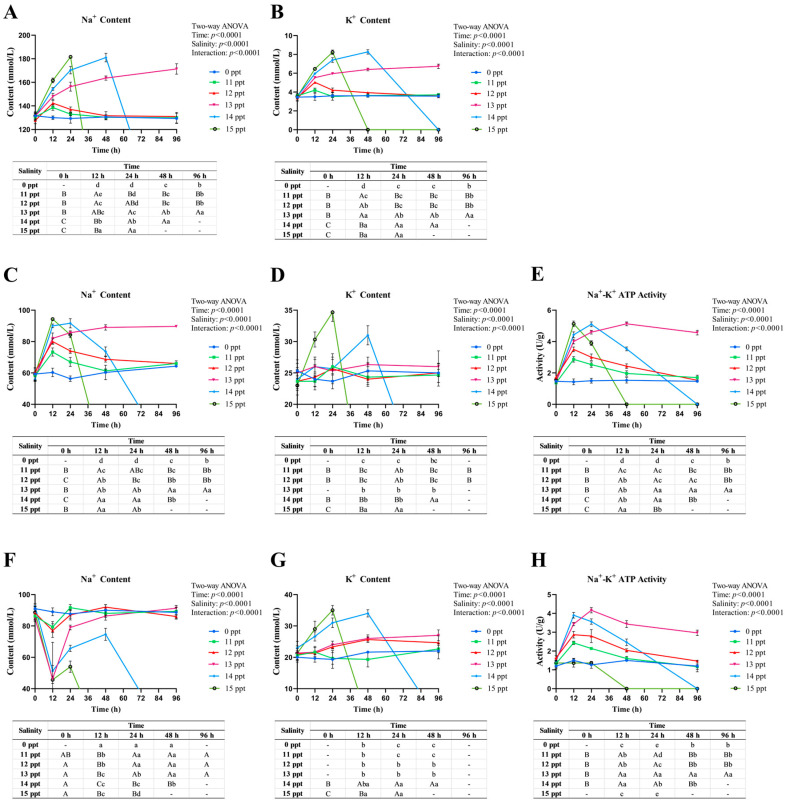
Ion content and Na^+^-K^+^ ATPase activity in osmoregulatory key tissues of *Perca schrenkii* under acute salinity stress. (**A**) Sodium (Na^+^) content in plasma. (**B**) Potassium (K^+^) content in plasma. (**C**) Na^+^ content in gill. (**D**) K^+^ content in gill. (**E**) Na^+^-K^+^ ATPase activity in gill. (**F**) Na^+^ content in kidney. (**G**) K^+^ content in kidney. (**H**) Na^+^-K^+^ ATPase activity in kidney. The *X*-axis represents exposure time (h), and the *Y*-axis represents ion content (mmol/L) or enzyme activity (U/g). Different colors denote salinity groups (0, **11**, **12**, **13**, **14**, and **15** ppt). Significant differences are marked by lowercase letters (*p* < **0.05**) for comparisons among salinity groups at the same time point, and uppercase letters (*p* < **0.05**) for comparisons among time points within the same salinity group. Two-way ANOVA results for time, salinity, and their interaction are shown in the top right corner of each subplot.

Plasma Na^+^ (Figure 3A) remained stable in **0–12** ppt groups, with transient increases at **12** h (**11–12** ppt) reflecting acute stress, followed by recovery. At **13** ppt, Na^+^ sustained elevation indicated prolonged compensation, while **14** ppt groups showed drastic surges due to regulatory failure. Plasma K^+^ (Figure 3B) stayed within **2.5–4.5** mmol/L in **0–12** ppt groups but progressively increased in **13** ppt groups, peaking at **9.0–11.0** mmol/L at **15** ppt (**96** h), signaling neuromuscular dysfunction.

Gill Na^+^ (Figure 3C) transiently increased in **11–12** ppt groups (**12** h) then recovered, while **13** ppt showed sustained elevation and ≥**14** ppt groups exhibited early peaks followed by declines. Gill Na^+^-K^+^ ATPase (Figure 3E) peaked at **12** h in **11**–**13** ppt groups (compensatory activation) but declined in ≥**14** ppt groups. Gill K^+^ (Figure 3D) remained stable except for late-stage increases in ≥**14** ppt groups.

Renal Na^+^ (Figure 3F) transiently decreased in **11–12** ppt groups then recovered, while ≥**14** ppt groups showed initial reduction followed by increases. Renal Na^+^-K^+^ ATPase (Figure 3H) followed a similar pattern to gill enzyme, with sustained activity in **13** ppt and decline in ≥**14** ppt groups. Renal K^+^ (Figure 3G) only increased in ≥**14** ppt groups at late time points.

Collectively, *P. schrenkii* exhibited reversible osmoregulation at ≤**12** ppt, prolonged compensation at **13** ppt, and irreversible failure at ≥**14** ppt, with **13** ppt as the critical threshold for osmoregulatory collapse.

##### Oxidative Stress of Acute Salinity Stress

**Figure 4:**
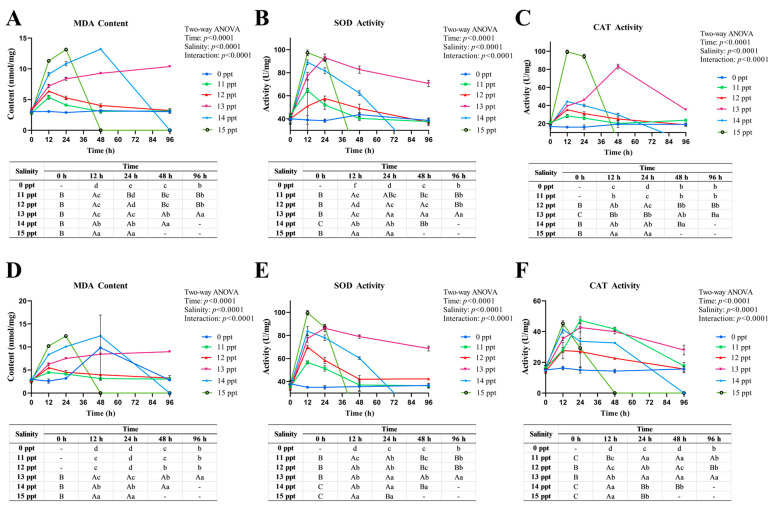
Oxidative stress responses in key antioxidant tissues of *Perca schrenkii* under acute salinity stress. (**A**) Malondialdehyde (MDA) content in gill. (**B**) Superoxide dismutase (SOD) activity in gill. (**C**) Catalase (CAT) activity in gill. (**D**) MDA content in intestine. (**E**) SOD activity in intestine. (**F**) CAT activity in intestine. The *X*-axis represents exposure time (h), and the *Y*-axis represents MDA content (nmol/mg prot), SOD activity (U/mg prot), or CAT activity (U/mg prot). Different colors denote salinity groups (0, **11**, **12**, **13**, **14**, and **15** ppt). Significant differences are marked by lowercase letters (*p* < **0.05**) for comparisons among salinity groups at the same time point, and uppercase letters (*p* < **0.05**) for comparisons among time points within the same salinity group. Two-way ANOVA results for time, salinity, and their interaction are shown in the top right corner of each subplot.

Acute salinity stress induced tissue-specific oxidative stress responses in *P. schrenkii*, characterized by dynamic changes in malondialdehyde (MDA) and antioxidant enzyme (SOD, CAT) activities in gill and intestine (Figure 4).

Gill MDA content (Figure 4A) transiently increased in **11–12** ppt groups at **12** h (compensatory oxidative damage) then recovered, while ≥**13** ppt groups showed sustained elevation, indicating severe lipid peroxidation. Gill SOD activity (Figure 4B) was activated in **11**–**13** ppt groups at **12** h but declined in ≥**14** ppt groups. Gill CAT activity (Figure 4C) followed a similar trend, with transient activation in **11**–**13** ppt and decline in ≥**14** ppt, reflecting impaired H_2_O_2_ scavenging.

Intestinal MDA content (Figure 4D) exhibited trends analogous to gill but with lower amplitude, showing transient increases in **11–12** ppt and sustained elevation in ≥**13** ppt. Intestinal SOD (Figure 4E) and CAT (Figure 4F) activities were activated in **11**–**13** ppt groups and declined in ≥**14** ppt groups, consistent with systemic oxidative damage progression.

Collectively, *P. schrenkii* displayed reversible oxidative stress at ≤**12** ppt, prolonged antioxidant compensation at **13** ppt, and antioxidant system collapse with severe oxidative damage at ≥**14** ppt. Gill, as the frontline tissue, showed more pronounced oxidative stress than the intestine, aligning with its direct exposure to salinity stress.

##### Immune Function of Acute Salinity Stress

**Figure 5:**
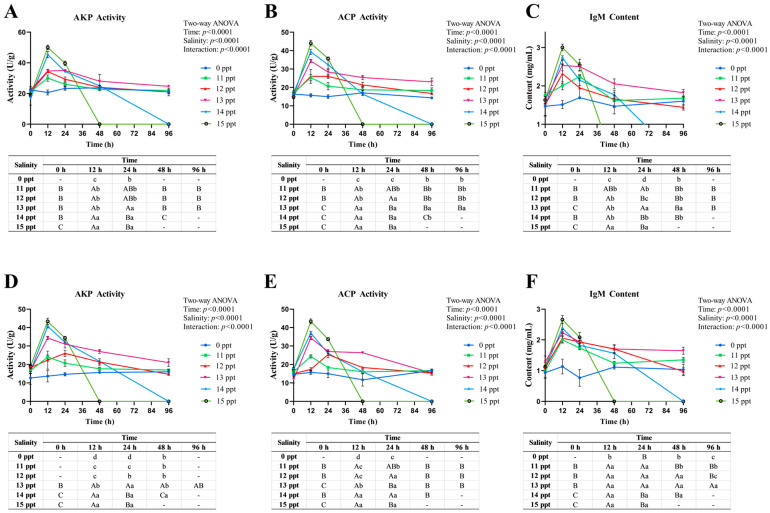
Immune regulatory responses in key immune tissues of *Perca schrenkii* under acute salinity stress. (**A**) Alkaline phosphatase (AKP) activity in liver. (**B**) Acid phosphatase (ACP) activity in liver. (**C**) Immunoglobulin M (IgM) content in liver. (**D**) AKP activity in kidney. (**E**) ACP activity in kidney. (**F**) IgM content in kidney. The *X*-axis represents exposure time (h), and the *Y*-axis represents enzyme activity (U/g) or IgM content (mg/mL). Different colors denote salinity groups (0, **11**, **12**, **13**, **14**, and **15** ppt). Significant differences are marked by lowercase letters (*p* < **0.05**) for comparisons among salinity groups at the same time point, and uppercase letters (*p* < **0.05**) for comparisons among time points within the same salinity group. Two-way ANOVA results for time, salinity, and their interaction are shown in the top right corner of each subplot.

Acute salinity stress induced dynamic immune regulatory responses in *P. schrenkii*, characterized by tissue-specific changes in immune enzyme (AKP, ACP) activities and immunoglobulin M (IgM) content in liver and kidney (Figure 5).

Liver AKP (Figure 5A) and ACP (Figure 5B) activities showed transient activation in low-salinity groups at **12** h followed by recovery, reflecting acute stress-induced immune activation. At **13** ppt, both enzymes sustained high activity at **96** h, indicating prolonged immune compensation. In high-salinity groups (≥**14** ppt), enzyme activities peaked at **12** h then declined, consistent with hepatic tissue damage. Liver IgM (Figure 5C) exhibited analogous trends: transient elevation in **11–12** ppt and sustained increase in **13** ppt, while ≥**14** ppt groups showed early peaks followed by decreases, reflecting impaired immunoglobulin synthesis.

Kidney AKP (Figure 5D) and ACP (Figure 5E) activities were activated in **11**–**13** ppt groups at **12** h, with **13** ppt maintaining sustained activity at **96** h. In ≥**14** ppt groups, activities peaked at **12** h then declined, indicative of renal immune dysfunction. Kidney IgM (Figure 5F) transiently increased in **11**–**13** ppt and decreased in ≥**14** ppt, aligning with systemic immune suppression.

Collectively, *P. schrenkii* displayed reversible immune activation at ≤**12** ppt, prolonged immune compensation at **13** ppt, and immune system collapse with tissue damage at ≥**14** ppt. The liver, as the central immune organ, exhibited more pronounced responses than the kidney, highlighting its pivotal role in salinity-induced immune regulation.

### 3.2. Effects of Chronic Salinity Stress on Perca schrenkii

#### 3.2.1. Osmotic Regulation of Chronic Salinity Stress

**Figure 6:**
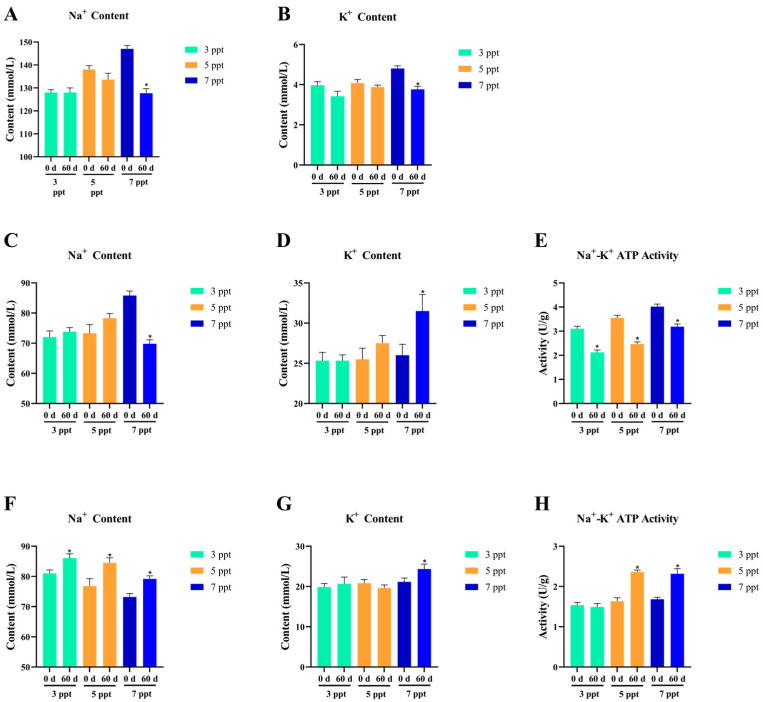
Osmotic regulation in key osmoregulatory tissues of *Perca schrenkii* under chronic salinity stress (**60** days). (**A**) Sodium (Na^+^) content in plasma. (**B**) Potassium (K^+^) content in plasma. (**C**) Na^+^ content in gill. (**D**) K^+^ content in gill. (**E**) Na^+^-K^+^ ATPase activity in gill. (**F**) Na^+^ content in kidney. (**G**) K^+^ content in kidney. (**H**) Na^+^-K^+^ ATPase activity in kidney. The *X*-axis represents salinity (3, 5, 7 ppt) with time points (0 d, **60** d). The *Y*-axis indicates: plasma Na^+^/K^+^ (mmol/L), gill/kidney Na^+^/K^+^ (mmol/L), and Na^+^-K^+^ ATPase activity (U/g). Different colors denote salinity groups (3, 5, 7 ppt). Asterisks (*) indicate significant differences between **60** d and 0 d at the same salinity (*p* < **0.05**).

Chronic salinity stress (3, 5, 7 ppt for **60** days) triggered adaptive osmoregulatory responses in *Perca schrenkii*, characterized by dynamic adjustments in ion content (Na^+^, K^+^) and Na^+^-K^+^ ATPase activity across plasma, gill, and kidney, ultimately achieving a new steady state after long-term acclimation (Figure 6).

Plasma Na^+^ (Figure 6A) exhibited salinity-dependent elevation at 0 d: 3 ppt, 5 ppt, and 7 ppt, reflecting initial osmotic stress. After **60** days, plasma Na^+^ decreased significantly (*p* < **0.05**) in the 7 ppt group and returned to near-baseline levels in **3–5** ppt groups, indicating efficient recovery of ion balance. Plasma K^+^ (Figure 6B) showed mild fluctuations at 0 d but no significant differences (*p* > **0.05**) between **60** d and 0 d across all salinities, with **60** d values stabilizing at **3.0–4.5** mmol/L—highlighting robust K^+^ homeostasis.

Gill Na^+^ (Figure 6C) increased at 0 d with rising salinity due to acute salt uptake. At **60** d, gill Na^+^ decreased significantly (*p* < **0.05**) in the 7 ppt group and approached baseline levels, reflecting adaptive ion excretion. Gill K^+^ (Figure 6D) remained stable at **25–30** mmol/L across all treatments, with no temporal differences, consistent with the gill’s primary role in Na^+^ transport. Gill Na^+^-K^+^ ATPase (Figure 6E) displayed a stress-acclimation pattern: 0 d activity increased with salinity to counter initial stress, then decreased significantly (*p* < **0.05**) at **60** d—downregulated to a balanced level for sustainable ion transport.

Renal Na^+^ (Figure 6F) showed an inverse trend to gill Na^+^ at 0 d: salinity elevation enhanced Na^+^ reabsorption, leading to lower renal Na^+^ content. At **60** d, renal Na^+^ increased significantly (*p* < **0.05**) in the 7 ppt group as reabsorption intensity diminished, returning to near-baseline levels. Renal K^+^ (Figure 6G) remained stable at **20–25** mmol/L across all treatments, emphasizing priority in K^+^ homeostasis. Renal Na^+^-K^+^ ATPase (Figure 6H) mirrored the gill enzyme’s adaptation: 0 d activity peaked at higher salinities to support Na^+^ reabsorption, then decreased significantly (*p* < **0.05**) at **60** d—aligning with reduced reabsorption demand post-acclimation.

Collectively, *P. schrenkii* demonstrated complete osmoregulatory acclimation to chronic low-salinity stress (**3–7** ppt). The initial stress-induced perturbations were reversed or stabilized after 60 days, with 7 ppt showing the most pronounced adjustments without ion dysfunction. This indicates salinities ≤ 7 ppt are long-term tolerable for *P. schrenkii*.

#### 3.2.2. Oxidative Stress of Chronic Salinity Stress

**Figure 7:**
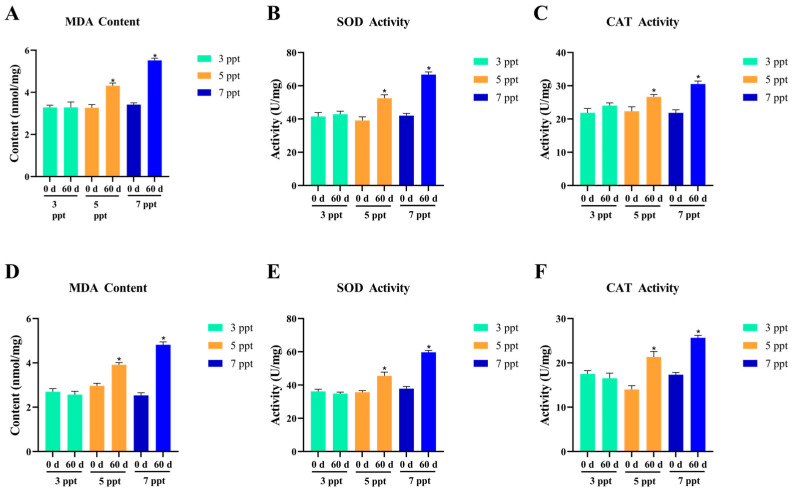
Oxidative stress responses in key antioxidant tissues of *Perca schrenkii* under chronic salinity stress (**60** days). (**A**) Malondialdehyde (MDA) content in gill. (**B**) Superoxide dismutase (SOD) activity in gill. (**C**) Catalase (CAT) activity in gill. (**D**) MDA content in intestine. (**E**) SOD activity in intestine. (**F**) CAT activity in intestine. The *X*-axis represents salinity (3, 5, 7 ppt) with time points (0 d, **60** d). The *Y*-axis indicates MDA content (nmol/mg prot), SOD activity (U/mg prot), or CAT activity (U/mg prot). Different colors denote salinity groups (3, 5, 7 ppt). Asterisks (*) indicate significant differences between **60** d and 0 d at the same salinity (*p* < **0.05**).

Chronic salinity stress (3, 5, 7 ppt for **60** days) induced adaptive oxidative stress responses in *Perca schrenkii*, characterized by tissue-specific changes in oxidative damage (MDA) and antioxidant enzyme (SOD, CAT) activities in gill and intestine, with successful acclimation observed at ≤7 ppt (Figure 7).

Gill MDA (Figure 7A) showed no significant difference at 3 ppt between **60** d and 0 d, indicating complete recovery of oxidative damage. At **5–7** ppt, MDA increased significantly (*p* < **0.05**) at 60 d compared to 0 d, reflecting mild, adaptive oxidative stress without irreversible damage. Gill SOD (Figure 7B) and CAT (Figure 7C) activities exhibited a stress-acclimation pattern: at 3 ppt, activities remained stable, consistent with basal antioxidant capacity. At **5–7** ppt, SOD and CAT increased significantly (*p* < **0.05**) at **60** d, representing sustained antioxidant activation to counteract mild oxidative stress.

Intestinal MDA (Figure 7D) followed a trend analogous to gill but with lower amplitude: no difference at 3 ppt, while **5–7** ppt showed significant increases (*p* < **0.05**) at **60** d, indicating mild oxidative stress localized to the gut. Intestinal SOD (Figure 7E) and CAT (Figure 7F) activities mirrored gill responses but with reduced magnitude: 3 ppt maintained basal levels, while **5–7** ppt showed significant activation (*p* < **0.05**) at **60** d, reflecting coordinated antioxidant defense across tissues.

Collectively, *P. schrenkii* exhibited adaptive oxidative stress responses under chronic low-salinity stress (**3–7** ppt). At 3 ppt, the antioxidant system returned to basal homeostasis; at **5–7** ppt, mild oxidative stress was countered by sustained antioxidant enzyme activation, with no signs of oxidative damage or enzyme dysfunction. This indicates that salinities ≤ 7 ppt are within the long-term tolerable range for *P. schrenkii*, with the antioxidant system fully capable of maintaining redox balance.

#### 3.2.3. Immune Function of Chronic Salinity Stress

**Figure 8:**
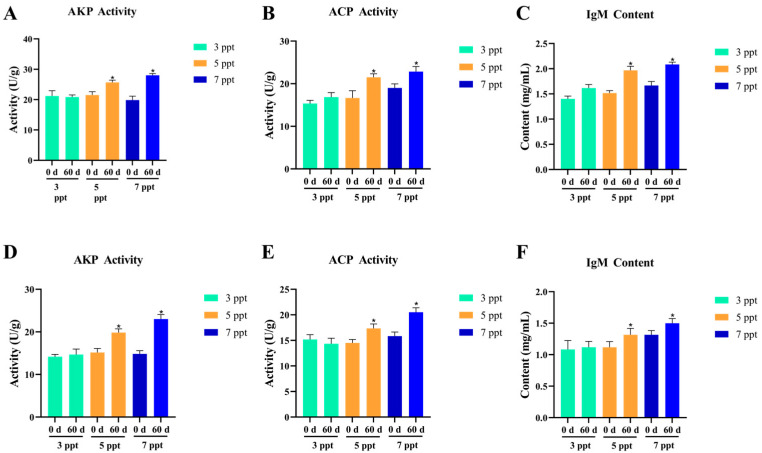
Immune regulatory responses in key immune tissues of *Perca schrenkii* under chronic salinity stress (60 days). (**A**) Alkaline phosphatase (AKP) activity in liver. (**B**) Acid phosphatase (ACP) activity in liver. (**C**) Immunoglobulin M (IgM) content in liver. (**D**) AKP activity in kidney. (**E**) ACP activity in kidney. (**F**) IgM content in kidney. The *X*-axis represents salinity (3, 5, 7 ppt) with time points (0 d, 60 d). The *Y*-axis indicates enzyme activity (U/g) or IgM content (mg/mL). Different colors denote salinity groups (3, 5, 7 ppt). Asterisks (*) indicate significant differences between 60 d and 0 d at the same salinity (*p* < **0.05**).

Chronic salinity stress (3, 5, 7 ppt for **60** days) induced adaptive immune regulatory responses in *Perca schrenkii*, characterized by tissue-specific changes in immune enzyme (AKP, ACP) activities and immunoglobulin M (IgM) content in liver and kidney, with successful immune homeostasis maintained at ≤7 ppt (Figure 8).

Liver AKP (Figure 8A) and ACP (Figure 8B) activities showed no significant difference at 3 ppt between 60 d and 0 d, indicating complete recovery of immune enzyme function. At 5–7 ppt, AKP and ACP increased significantly (*p* < **0.05**) at 60 d, reflecting mild, adaptive activation of lysosomal enzymes for enhanced foreign matter clearance. Liver IgM (Figure 8C) exhibited a consistent trend: no difference at 3 ppt, while **5–7** ppt showed significant increases (*p* < **0.05**) at 60 d, representing sustained humoral immune activation to maintain defense capacity under mild stress.

Renal AKP (Figure 8D) and ACP (Figure 8E) activities mirrored hepatic responses but with reduced magnitude: no difference at 3 ppt, while **5–7** ppt showed significant activation (*p* < **0.05**) at **60** d, reflecting coordinated lysosomal enzyme activity across tissues for immune surveillance. Renal IgM (Figure 8F) followed the same pattern as liver IgM but with lower amplitude: no difference at 3 ppt, and significant increases (*p* < **0.05**) at **5–7** ppt at **60** d, ensuring intact humoral immune barriers in the renal microenvironment.

Collectively, *P. schrenkii* exhibited adaptive immune regulation under chronic low-salinity stress (**3–7** ppt). At 3 ppt, the immune system returned to basal homeostasis; at **5–7** ppt, mild stress triggered sustained activation of immune enzymes and humoral immunity, with no signs of immune suppression or overactivation. This indicates that salinities ≤ 7 ppt are within the long-term tolerable range for *P. schrenkii*, with the immune system fully capable of maintaining defense function and homeostasis.

## 4. Discussion

Salinity stress—whether acute or chronic—profoundly shapes the survival and physiological homeostasis of euryhaline fish, with distinct response mechanisms underlying tolerance to extreme vs. sub-lethal conditions [30,31,32,33]. Globally, freshwater scarcity drives the exploration of saline-alkaline aquaculture, while chloride-type saline-alkaline water represents a critical underutilized resource [14,34]. *Perca schrenkii*, endemic to the Balkhash Lake basin with distribution in brackish-freshwater ecotones, yet its physiological adaptation mechanisms to chronic low-salinity stress remain elusive. Two core questions limit the commercialization of *Perca schrenkii*: (1) What is its acute high-salinity tolerance threshold? (2) Can it adapt to long-term low-salinity conditions?

This study addressed both questions through two complementary experiments: (1) The acute high-salinity stress experiment that identified a **96** h median lethal concentration (LC_50_) of **12.396** ppt and a safe concentration (SC) of **3.7188** ppt, with fish exhibiting a compensation-failure response: reversible adaptation at ≤**12** ppt, prolonged compensation at **13** ppt, and irreversible physiological collapse at ≥**14** ppt. (2) The chronic low-salinity acclimation experiment that demonstrated complete adaptability to ≤7 ppt, via a mild activation-steady state pattern across osmotic, antioxidant, and immune systems. Together, these findings form a threshold-adaptation framework: *P. schrenkii* tolerates acute salinity up to **12** ppt (LC_50_) and adapts long-term to ≤7 ppt, with the SC aligning with the non-stress adaptation point (3 ppt) observed in chronic experiments—closing the gap between acute toxicity and chronic cultivation practice.

### 4.1. The Defined Salinity Threshold and Its Ecological Implications

The accurately determined **96** h LC_50_ of **12.396** ppt positions *P. schrenkii* as a species with superior innate salinity tolerance compared to many traditional freshwater aquaculture species [35]. For instance, the **96** h LC_50_ of Nile tilapia (*Oreochromis niloticus*), a widely cultivated euryhaline fish, ranges from **10.5** to **11.8** ppt under similar chloride-type saline stress [35]. More importantly, this value is notably higher than that reported for its congener, *Perca fluviatilis* [25,36]. This interspecific discrepancy is ecologically significant. The Ili River basin, the native habitat of *P. schrenkii*, is characterized by fluctuating hydrochemistry and seasonal salinity increases, particularly in its delta region [37,38,39]. Therefore, our laboratory-derived LC_50_ likely reflects an evolutionary adaptation to its naturally unstable habitat, providing a physiological basis for its observed dominance in oligohaline reaches. The concept of a critical threshold is further reinforced by our behavioral and physiological data, which collectively identify **13** ppt as the pivot point between compensatory homeostasis and irreversible failure.

### 4.2. Osmoregulatory Strategy: From Compensation to Collapse

The osmoregulatory response of *P. schrenkii* exemplifies a classic, yet finely tuned, teleost strategy under osmotic stress. The transient increase in gill and renal Na^+^-K^+^-ATPase activity in the **11**–**13** ppt groups represents a critical compensatory effort to counteract passive ion influx and maintain plasma homeostasis [40]. This energetic investment in ion regulation is a survival tactic [41,42,43]. However, the subsequent decline in enzyme activity at ≥**14** ppt is not merely a “failure” but likely a consequence of multiple cascading insults: severe ion dysregulation leading to cellular damage, ATP depletion due to unsustainable energetic demands, and potentially, oxidative inactivation of enzyme proteins [42]. The parallel surge in plasma Na^+^ and K^+^ at lethal salinities indicates a complete breakdown of the osmoregulatory interface, culminating in neuromuscular dysfunction and death. This pattern underscores that the cost of osmoregulation under extreme stress becomes prohibitive, leading to systemic collapse.

### 4.3. Oxidative Stress and Immune Response: An Integrated Defense Network

A key finding of our study is the tight coupling between osmotic imbalance and secondary oxidative and immune stress. The initial upregulation of SOD and CAT in the gill and intestine at sublethal salinities (**11**–**13** ppt) demonstrates a proactive antioxidant defense against salinity-induced reactive oxygen species (ROS) [44,45]. The gill, as the primary site of ion and gas exchange, exhibited more pronounced oxidative stress than the intestine, aligning with its direct and extensive contact with the external medium [46,47]. The subsequent decline in antioxidant capacity at lethal salinities signifies the overwhelming of this defense system, leading to the observed accumulation of MDA, a marker of irreversible lipid peroxidation [48,49].

Similarly, the immune system displayed a biphasic response. The activation of AKP, ACP, and IgM in liver and kidney under moderate stress suggests an enhanced state of immune vigilance [50,51]. The subsequent suppression of these physiological parameters under acute lethal salinity stress reflects a systemic integrated physiological collapse, wherein energy allocation is redirected away from non-essential homeostatic processes (e.g., adaptive immunity) toward emergency compensatory mechanisms dedicated to preserving core physiological homeostasis [52,53,54]. Under chronic stress, the sustained, yet stable, elevation of these markers at **5–7** ppt indicates a successful re-allocation of resources, establishing a new, sustainable homeostatic set-point without apparent damage.

### 4.4. Acclimation Potential and Aquaculture Relevance

The chronic experiment validates the aquaculture potential of *P. schrenkii*. The fact that fish fully acclimated to 7 ppt salinity over **60** days, with osmoregulatory, antioxidant, and immune parameters stabilizing at new steady states, is of paramount practical importance. The initial stress responses observed at Day 0 gave way to acclimation by Day **60**, particularly the normalization of plasma ions and the refined modulation of Na^+^-K^+^-ATPase. This downregulation after acclimation is a hallmark of efficient adaptation, reducing the metabolic cost of osmoregulation and potentially freeing energy for growth. Our findings suggest that salinities up to 7 ppt are not only survivable but likely sustainable for long-term cultivation, with the safe concentration (**3.72** ppt) providing a conservative starting point for stocking.

### 4.5. Limitations and Future Perspectives

While our use of NaCl elegantly isolated the osmotic component of chloride-type saline-alkaline water, natural environments present a more complex ionic matrix (e.g., presence of Ca^2+^, Mg^2+^, SO_4_^2−^). Future studies should investigate whether these ions have synergistic or antagonistic effects on salinity tolerance. Furthermore, our study lays the physiological groundwork but calls for molecular validation. Transcriptomic and proteomic analyses are needed to unravel the precise regulatory networks governing ion transport, antioxidant defense, and immune modulation during acclimation.

## 5. Conclusions

This study moves beyond merely defining the salinity tolerance of *Perca schrenkii* and establishes a mechanistic framework for its adaptation to chloride-type saline stress. We conclude that: (1) *P. schrenkii* possesses a high innate salinity tolerance (**96** h LC_50_ = **12.396** ppt), with **13** ppt serving as a critical threshold beyond which coordinated physiological failure occurs across osmoregulatory, antioxidant, and immune systems. (2) The species exhibits remarkable long-term acclimation capacity, maintaining full physiological homeostasis at salinities up to 7 ppt. This acclimation is characterized by a reduction in metabolic costs associated with osmoregulation and the establishment of a new, stable equilibrium in oxidative and immune status. (3) The determined safe concentration (**3.72** ppt) and the demonstrated tolerance up to 7 ppt provide clear, evidence-based guidelines for the cultivation of *P. schrenkii* in saline-alkaline waters, paving the way for a sustainable aquaculture expansion in water-scarce regions.

## Figures and Tables

**Table 1:** Measurement methods for behavioral indicators of *Perca schrenkii* under acute salinity stress.

Indicator Category	Specific Indicator	Measurement Method
Swimming behavior	Swimming speed	Record the distance swam by the fish in **30** s, calculate mean speed (cm/s)
	Abnormal swimming pattern ratio	Statistic the duration of abnormal behaviors (lateral swimming, side-lying) in **10** min, calculate the proportion of abnormal duration to total observation time (%)
Respiratory behavior	Gill cover opening frequency	Count the number of gill cover openings in 5 min, convert to frequency per minute (times/min), take the mean of 3 counts
Stress avoidance behavior	Jumping times	Count the number of times the fish leaps out of the water within 1 h
	Rubbing frequency	Count the number of times the fish rubs its body against the tank wall within 1 h

**Table 2:** Detection methods and instrumentation for physiological and biochemical indicators of *Perca schrenkii* in salinity stress experiments.

Indicator Category	Indicators	Detection Method	Instrument Model
Ion balance	Na^+^, K^+^	Flame atomic absorption spectrophotometry	PerkinElmer AA800 PerkinElmer Inc., located in Waltham, MA, USA
Osmoregulation	Na^+^-K^+^-ATPase	Colorimetry	TM FC Microplate Reader Thermo Fisher Scientific Inc., located in Waltham, MA, USA
Oxidative stress	CAT, SOD	Colorimetry	TM FC Microplate Reader
	MDA	TBA method	TM FC Microplate Reader
Immune function	AKP, ACP	Colorimetry	TM FC Microplate Reader
	IgM	ELISA	BioTek ELx800 Microplate Reader BioTek Instruments, Inc., located in Winooski, VT, USA

Abbreviations: CAT: Catalase, SOD: Superoxide Dismutase, MDA: Malondialdehyde, AKP: Alkaline Phosphatase, ACP: Acid Phosphatase, IgM: Immunoglobulin M.

## Data Availability

The datasets generated and analyzed during the current study are available from the corresponding author on reasonable request.
